# Pancréatite aigüe idiopathique du post-partum: difficultés diagnostiques (à propos d’un cas)

**DOI:** 10.11604/pamj.2022.41.48.31342

**Published:** 2022-01-18

**Authors:** Abderrahmane Jallouli, Hicham Baba, Ahmed Zeroual, Mohamed Es-Said Ramraoui, Ahmed Elguazzar, Mohammed Lahkim, Ahmed El Khader, Rachid El Barni

**Affiliations:** 1 Service de Chirurgie Générale, Hôpital Militaire Avicenne de Marrakech, Marrakech, Maroc,; 2 Faculté De Médecine et de Pharmacie, Université Cadi Ayyad de Marrakech, Maroc

**Keywords:** Pancréatite aigüe, post-partum, idiopathique, cas clinique, Acute pancreatitis, postpartum, idiopathic, case report

## Abstract

L´installation d´une pancréatite aigüe en post-partum ou durant la grossesse est rare. La pathologie biliaire constitue de loin la cause la plus fréquente, suivie d´hypertriglycéridémie. Nous rapportons à travers cette observation le cas d´une pancréatite aigüe survenant 4 semaines en post-partum chez une patiente de 19 ans, révélée par des épigastralgies transfixiantes à irradiation dorsale, avec une lipasémie à 36 fois la normale, et dont le bilan étiologique était négatif. Le diagnostic précoce de cette entité constitue un défi au clinicien vu sa rareté et ses multiples diagnostics différentiels.

## Abstract (en)

Acute pancreatitis during the postpartum period or during pregnancy is rare. Biliary disease is the most common cause of acute pancreatitis, followed by hypertriglyceridemia. We here report the case of a 19-year-old patient with acute pancreatitis occurring 4 weeks after delivery and revealed by epigastralgia radiating to the back, lipasemia more than 36 times the normal limit and etiologic investigation that gave negative results. Early diagnosis of acute pancreatitis during the postpartum period is a challenge for clinicians because it is rare and differential diagnosis includes multiple disorders.

## Introduction

L´installation d´une pancréatite aigüe en post-partum ou durant la grossesse est rare, son incidence est plus importante en 3^e^trimestre, et faible en post-partum [1-3]. La pathologie biliaire constitue la cause la plus fréquente, suivie de l´hypertriglycéridémie [4]. Le diagnostic précoce constitue un défi au clinicien vu sa rareté et ses diagnostics différentiels. Le pronostic est lié à la qualité et la précocité de la prise en charge thérapeutique. Nous rapportons à travers cette observation le cas d´une pancréatite aigüe survenant 4 semaines en post-partum dont le bilan étiologique s´est révélé négatif.

## Patient et observation

**Information de la patiente:** il s´agit d´une patiente de 19 ans, primigeste et primipare, sans antécédents pathologiques particuliers, qui a présenté 4 semaines après son accouchement par césarienne des épigastralgies d´installation brutale, intenses, transfixiantes et à irradiation dorsale, associées à des nausées et des vomissements évoluant dans un contexte de sensations fébriles.

**Résultats cliniques:** l´examen clinique a objectivé une patiente consciente, stable sur le plan hémodynamique et respiratoire (fréquence cardiaque= 94 battements par minute, fréquence respiratoire= 22 cycles par minute, tension artérielle=138/80 mmHg, saturation en oxygène = 95%), apyrétique (température =36,7°C) et un score de SIRS (syndrome de réponse inflammatoire Systémique) à l´admission à 3. L´examen abdominal a montré une sensibilité épigastrique, sans autres anomalies décelables au reste de l´examen somatique.

**Démarche diagnostique:** le bilan biologique a révélé une lipasémie à 2200 UI/l (36 fois la normale), une hyperleucocytose à 22210/μl à prédominance neutrophiles (19290μ/l), une protéine C réactive (CRP) à 59 mg/l, une cytolyse hépatique (Aspartate aminotransférase (ASAT)= 321 U/l et Alanine aminotransférase (ALAT)= 464 U/l), ainsi qu´une cholestase biologique (Bilirubine totale= 132 μmol/l, Bilirubine conjuguée= 83,5 μmol/l, Phosphatases alcalines (PAL)= 240 U/l, Gamma glutamyl transférase (GGT)= 792 U/l), le reste du bilan biologique était sans anomalies. La tomodensitométrie (TDM) abdominale réalisée 72 heures après le début de la symptomatologie a montré un aspect de pancréatite aigüe stade E de Balthazar associée à des coulées de nécrose pancréatique. L´enquête étiologique a écarté la notion d´alcoolisme, de prise médicamenteuse particulière ou de cas similaires dans la famille, l´échographie abdominale a objectivé une vésicule biliaire alithiasique avec absence de dilatation des voies biliaires intra et extra hépatiques, la BILI-IRM n´a pas montré également d´obstacle au niveau des voies biliaires avec une vésicule biliaire de morphologie normale sans calculs, le bilan lipidique était normal (triglycérides à 1,10 g/l, cholestérol total à 1,12 g/l, cholestérol LDL à 0,74 g/l), la calcémie corrigée était à 2,53 mmol/l, le bilan immunologique ainsi que les sérologies virales (HIV, HVA, HVB, HVC, CMV, EBV) étaient négatives. Devant la négativité du bilan étiologique, le diagnostic de pancréatite aigüe idiopathique a été retenu.

**Intervention thérapeutique:** la prise en charge thérapeutique a comporté l´hospitalisation au service de chirurgie viscérale, l´arrêt d´alimentation entérale remplacée par une alimentation parentérale, une analgésie (paracétamol et néfopam), des antispasmodiques, et des inhibiteurs de la pompe à protons.

**Suivi et résultats:** l´évolution a été marquée par une amélioration clinico-biologique (SIRS à 0, CRP à 9 et leucocytes à 10850). La patiente a été déclarée sortante 14 jours après son admission.

**Consentement éclairé:** la patiente a déclaré son consentement librement et de façon éclairée, afin de permettre la réalisation et la publication de ce manuscrit.

## Discussion

La survenue d´une pancréatite aigüe durant la grossesse ou en post-partum est une situation particulière et rare, son incidence est de 1 à 14 cas pour 10.000 naissances [1, 5]. Elle se manifeste le plus souvent au cours du troisième trimestre, et plus rarement en post-partum [1-3]. Son diagnostic n´est pas facile vu ses diagnostics différentiels multiples: HELLP Syndrome, cholécystite aigüe, colique hépatique, appendicite aigue [1].

Les lithiases biliaires constituent l´étiologie la plus fréquente de pancréatite aigüe durant la grossesse et en post-partum (70%) [1], elles sont dues principalement à l´effet de la progestérone ; qui aboutit à une hypotonie des voies biliaires et une hypertonie du sphincter d´Oddi [6]. L´hypertriglycéridémie est la deuxième cause en termes de fréquence ; elle est responsable le plus souvent d´un tableau de pancréatite aigüe grave, des taux élevés de triglycérides (entre 750 à 1000 mg/dl) sont retrouvés chez 4 à 6% des cas durant le 2^e^et 3^e^trimestre, avec un taux de mortalité fœtale important (37%) [7]. La pancréatite aigüe associée à une hypertension gravidique est une situation rare dont l´évolution est défavorable [4]. Les autres étiologies sont similaires à ceux des pancréatites aigues du reste de la population: consommation excessive d´alcool, hypercalcémie, infection, auto-immune, traumatique, iatrogène... Cependant, il n´existe jusqu´à là aucun lien de causalité directe entre la grossesse elle-même et la survenue de pancréatite aigüe [4].

La pancréatite aigüe en post-partum garde les mêmes caractéristiques cliniques, biologiques et radiologiques; il s´agit souvent d´épigastralgies intenses transfixiantes avec irradiation dorsale, associées à des nausées, vomissements et fièvre, L´examen clinique objective une sensibilité voire défense épigastrique, une diminution des bruits hydroaériques par iléus reflexe, le signe de Murphy peut être présent. Le diagnostic est confirmé par le dosage de la lipasémie; il est supérieur à 3 fois la normale. On constate également une hyperleucocytose et une élévation des enzymes hépatiques et de la CRP. Les examens radiologiques (Echographie abdominale, TDM abdominale et IRM) permettent d´évaluer la sévérité de la pancréatite aigüe, et de rechercher l´étiologie [4]. La prise en charge thérapeutique de pancréatite aigüe du post-partum repose sur les recommandations classiques de toute pancréatite: mise au repos du tube digestif, réhydratation parentérale, analgésie, antibiothérapie et drainage des coulées de nécroses infectées. Le traitement étiologique fait appel à une cholécystectomie dans les cas de pancréatites biliaires, et un régime pauvre en lipides associé aux hypolipémiants pour les pancréatites secondaires à une hypertriglycéridémie [8]. Le pronostic de cette entité a connu une importante amélioration grâce à la disponibilité des différents outils biologiques (lipasémie) et radiologiques (échographie et scanner abdominal) qui permettent un diagnostic précoce et une prise en charge adaptée [5].

## Conclusion

La pancréatite aigüe du post-partum est rare, son diagnostic repose sur un faisceau d´arguments anamnestiques et cliniques, et une confirmation biologique. L´étiologie biliaire est la cause la plus fréquente suivie de l´hypertriglycéridémie. Un diagnostic précoce permet une prise en charge adéquate et par conséquence un meilleur pronostic.
